# Gender differences in face pareidolia: The effect of cognitive style and judgment criteria

**DOI:** 10.1177/20416695251364206

**Published:** 2025-08-25

**Authors:** Hao Wang, Zhigang Yang

**Affiliations:** 1 College of Education, 56667 Hebei University, China; 2 Handan Preschool Teachers College, China

**Keywords:** face pareidolia, gender differences, global-local processing, facial configuration processing

## Abstract

Face pareidolia refers to perceiving facial features on inanimate objects. Previous studies have identified gender differences in pareidolia, but the factors behind these differences remain unclear. This study examined potential influences, including task requirement, low-frequency information encoding ability, and cognitive style. University student participants reported what they saw in face-like object images and rated their face-likeness. A delayed matching task with blurred faces assessed encoding ability, and the Navon task examined cognitive style. Results showed that gender differences were influenced by task demands: women were more likely than men to perceive faces in objects, and this was not related to facial configuration processing. Additionally, a global processing tendency predicted higher pareidolia in women but not in men. Our findings suggest that gender differences in pareidolia are shaped by judgment criteria, with women adopting more relaxed criteria. This research contributes to understanding gender differences in social cognition.

## How to cite this article

Wang, H., & Yang, Z. (2025). Gender differences in face pareidolia: The effect of cognitive style and judgment criteria. *i-Perception*, *16*(4), 1–15. https://doi.org/10.1177/20416695251364206

## Introduction

Faces are the most important social stimulus encountered in daily life. Humans need to quickly detect and recognize a face among other surrounding stimuli. Through evolution, humans and other primates have been equipped with this extraordianary faculty. Humans can detect faces in less than 50 ms (Caharel et al., 2014; Crouzet et al., 2010; Taubert et al., 2011). However, this efficient processing can lead to occasional misidentification. People sometimes perceive faces on inanimate objects or random patterns (Rieth et al., 2011), such as on clouds, tree trunks, and the surface of the moon. This illusion, which is referred to as face pareidolia, reflects the high sensitivity of the visual system to face-like structures. Misjudgement is necessary for social animals, as it increases the likelihood of perceiving new social interactions. The benefits of quickly discovering others’ faces in the surrounding environment outweigh the costs of misidentifying objects as faces (Verpooten & Nelissen, 2010).

Recently, several studies have found gender differences in face pareidolia, with women more likely than men to perceive objects as faces (Pavlova et al., 2020; Pavlova, Scheffler et al., 2015; Proverbio & Galli, 2016). Pavlova, Scheffler et al. (2015) found that women were more likely than men to spontaneously find faces in Arcimboldo-like patterns composed of food and plates. Proverbio and Galli (2016) used daily face-like objects as stimuli and found that women had higher scores on rating the degree of objects as face-like than men. However, some studies have found no such gender differences (Pavlova, Heiz et al., 2018; Wardle et al., 2022) or found gender differences only in the face inversion condition (Pavlova et al., 2020). Therefore, the specific mechanisms of gender differences in face pareidolia and whether the differences in previous study results are affected by task requirements remain unclear.

Previous studies have revealed gender differences in real facial processing, with women having superior ability in facial configuration processing, facial identity discrimination (McBain et al., 2009; McClure, 2000; Rehnman & Herlitz, 2006), and recognition of facial expression (McClure, 2000). McBain et al. (2009) demonstrated that women exhibited superior processing ability for line and real faces masked by noise, suggesting that women may have an advantage in processing low-frequency information, which is the main component of the configuration structure of faces (Keil, 2008). This ability has been associated with detecting faces from random patterns (Hansen et al., 2010; Liu et al., 2014; Tsao & Livingstone, 2008). The recognition of a stimulus as a face depends on spatial relationships among features (first-order configuration), particularly the positions of the eyes and mouth (Dakin & Watt, 2009; Omer et al., 2019). The global configuration (T-shaped) underlies the detection of face-like structures (Tsao & Livingstone, 2008), and differences in face pareidolia may demonstrate natural variations in the ability to detect this global configuration. Although the ability to process face structures can predict facial recognition (Richler et al., 2011; Wang et al., 2012), whether this ability influences face pareidolia remains unclear.

Moreover, perceiving a scrambled object as a face implies processing it as a whole (Caharel et al., 2013; Tsao & Livingstone, 2008); suggesting that pareidolia is influenced by the viewer's cognitive style. Gender differences in the global-local processing bias have previously been demonstrated (Pletzer et al., 2017; Razumnikova & Volf, 2012; Roalf et al., 2006) and may account for gender differences in face pareidolia. Global processing is a prerequisite for scrambled objects to be viewed as faces (Boccia et al., 2014; Caharel et al., 2013; Pavlova et al., 2020); however, studies have found that men are more inclined to engage in global processing, whereas women tend to focus on local feature processing. Nevertheless, previous research suggests that if global processing promotes pareidolia, men should perceive more faces than women,which contradicts the results of studies on face pareidolia. Therefore, further research is needed to understand the effects of cognitive style on gender differences in pareidolia.

In summary, the specific mechanism that causes gender differences in face pareidolia requires further investigation. Therefore, this study investigated factors affecting gender differences in face pareidolia, including task requirements, low-frequency information encoding ability, and cognitive style regarding common objects.

## Materials and Methods

### Participants

The recruitment of participants occurred in two stages. Initially, 70 university students (33 men and 37 women) participated in Study 1 (Tasks 1–3). The male participants were aged between 18 and 27 years (*M*_age_: 20.21, *SD* = 2.20), and the female participants were aged between 18 and 26 years (*M*_age_: 19.44, *SD* = 2.36).

Subsequently, an additional 21 university students were recruited to join the 70 participants from Study 1, resulting in a total of 91 participants (45 men and 46 women) in Study 2 (Tasks 2 and 4). All participants completed the tasks 2 following the same procedure as in Study 1. The sample size was determined with reference to the study by Pletzer et al. (2017). The male participants were aged 18–26 years (*M* = 20.11, *SD* = 2.10), and the female participants were aged 18–27 years (*M* = 19.34, *SD* = 2.12).

Participants were recruited using an on-campus online system and received academic credits in return. They all had normal or corrected-to-normal visual ability, reported no history of neurological illness or drug abuse, and were right-handed. All experiments were conducted after obtaining written informed consent from each participant. Participation was voluntary, and the data were processed anonymously. The experiment was approved by the Ethics Committee of Hebei University.

### Materials

**Figure 1.**
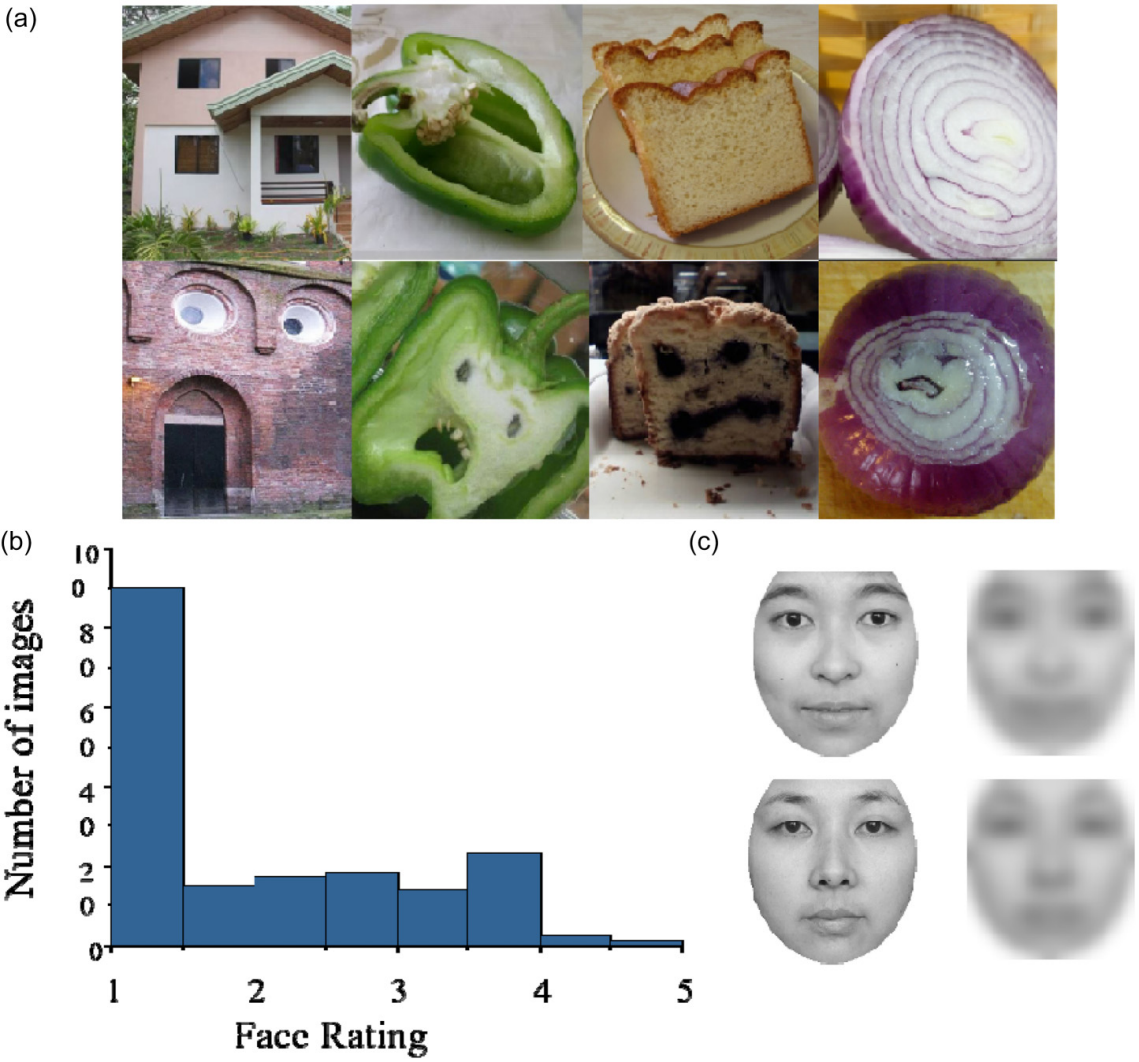
(a) Examples of common object images (below) and face-like object images (above); (b) average face-rating score (*x*), and frequency distribution of face ratings for all images; (c) examples of clear faces (left) and corresponding blurred faces (right) used in the blurred face delayed matching task.

We used 224 full-colour photographs of everyday objects—sourced via Internet image searches—as stimuli to elicit face pareidolia. All images were cropped to 530 × 480 pixels. Forty-two students (20 men and 22 women) who did not participate in the formal experiments were asked to rate the degree of face-likeness of all images on a 5-point rating scale (1 = non-face-like; 5 = face-like). Images with an average score of less than 1.5 were classified as common objects, and those with a score greater than 1.5 were classified as face-like objects. A total of 112 face-like and 112 non-face-like objects were included as stimuli (examples are presented in Figure 1a). Twenty-two students (9 men and 13 women) were asked to rate the perceived affective arousal intensity of 112 face-like objects on a 5-point scale. No significant differences were observed between genders, *t*(111) = 1.846, *p* = .068. Twelve images varying in face-likeness (*M* = 2.94, *SD* = 0.47; range = 2.19–3.73) were used in Task 1. To ensure consistency in perceived face-likeness, 17 students (7 men and 10 women) were asked to sort the images in ascending order of face-likeness, from least to most face-like. Kendall's concordance coefficient was calculated (*W* = 0.502, *χ*^2^ = 93.874, *p* < .01) and indicated high interrater consistency.

A total of 184 images were used for Task 2 (92 face-like objects), four of which were used for the practice session (two objects, two face-like objects). The remaining 180 images, half of which were face-like, were used for the formal session. The average face ratings for all images are shown in Figure 1b.

Low-frequency face images of 32 men and 32 women with neutral facial expressions were obtained from the native Chinese Facial Affective Picture System (CFAPS Gong, Huang, Wang & Luo, 2011). First, we used Photoshop 6.0 to convert the original face images (all 530 × 480 pixels) into grayscale. Each image was then cropped using an elliptical mask on a gray background, resized to 248 × 268 pixels, retaining only facial information while eliminating other features. Then, we applied the SHINE toolbox in MATLAB (R2019b) to standardize all images, ensuring consistent brightness and contrast across them. A Gaussian blur value of 15 was applied to each image to obtain blurred faces in which only configural information was retained (Schwaninger, 2003). Finally, 64 clear faces and 64 blurred faces were obtained, eight of which were used for the practice session (four clear and four blurred faces), and 120 were used for the formal experiment (60 blurred faces). Examples are shown in Figure 1c.

Navon-style compound letters were used for Task 4. Large letters were constructed from small letters. The target condition consisted of global letters that were either Es composed of local Hs, Ls, and Ts or global Hs, Ls, and Ts composed of local Es. In the non-target condition, the global letters were either Fs composed of local Hs, Ls, and Ts or global Hs, Ls, and Ts composed of local Fs. All stimuli consisted of a gray background and black letters, with all elements identical except for the letters. The large letters (composed of multiple small letters) subtended a visual angle of 4°×6°, and the small individual letters subtended a visual angle of 0.7°×1°. To ensure that the global and local processing tendencies between individuals were differentiated (Lamb & Robertson, 1989).

### Task and Procedure

Task 1 was a spontaneous reporting task. All participants were seated in a light- and sound-attenuated room. They sat comfortably on a chair with their face positioned 53 cm away from a 14-in. computer screen with a 60-Hz refresh frequency. They were instructed to focus on a fixation in the center of the screen. The experiment was programmed using E-Prime 2.0 (Psychology Software Tools, Inc., Pittsburgh, Pennsylvania, USA). Participants were presented with a set of images in ascending order of face-likeness, ranging from the least recognizable (1) to the most recognizable face (12). In each trial, participants were asked to briefly describe what they saw, write it down on an answer sheet, and then press the spacebar to switch to the next image. There was no time limit for this task. Subsequently, the contents of the participants’ descriptions were categorized into face reports (1) and non-face reports (0), according to the classification criteria presented by Pavlova, Scheffler et al. (2015). The participants described either a common object (non-face report: house, box, mop, pepper) or a face (face report: man in the hat, alien). Finally, the percentage of face reports for each participant was calculated.

Task 2 was a face-likeness rating task. After finishing Task 1, all participants were given Task 2 in the same environment. They were asked to rate the face-likeness of the object in each picture on a five-point rating scale (1 = *non-face-like*, 5 = *face-like*). The task had two blocks, each containing 90 trials, including 45 common objects and 45 face-like objects (15 for each of the three grades). Each trial was initiated by a black “+” sign displayed at the center of the gray screen for 500 ms, followed by the stimulus. The stimuli were terminated by the participants’ keypresses. After a blank gray screen was presented for 700 ms, the next trial was conducted. Before the formal experiment, the participants completed a pretest to familiarize themselves with the task (two objects and two face-like objects).

Task 3 is a delayed matching task of blurred faces. The experimental paradigm was adapted from the facial identity discrimination task by McBain et al. (2009), which was used to assess participants’ ability to process configural face information under low spatial frequency conditions. The participants who participated in the previous two tasks continued with Task 3 in the same environment (location, equipment). Participants were asked to match a delayed blurred face with a corresponding clear face. The experiment contained 60 trials, and each trial was initiated by a black “+” sign displayed at the center of the gray screen for 500 ms. A clear face appeared for 800 ms, followed by a blank screen for 800 ms. Subsequently, two blurred faces appeared on the left and right sides of the screen, one of them being a blurred version of the clear face that was initially presented. Participants were instructed to indicate which of the two blurred faces matched the one that was presented. The stimuli were terminated by the participant's key-press response, followed by a blank white screen for 100 ms and then the next trial. Before the formal experiment, the participants completed a practice session to familiarize themselves with the procedure.

**Figure 2.**
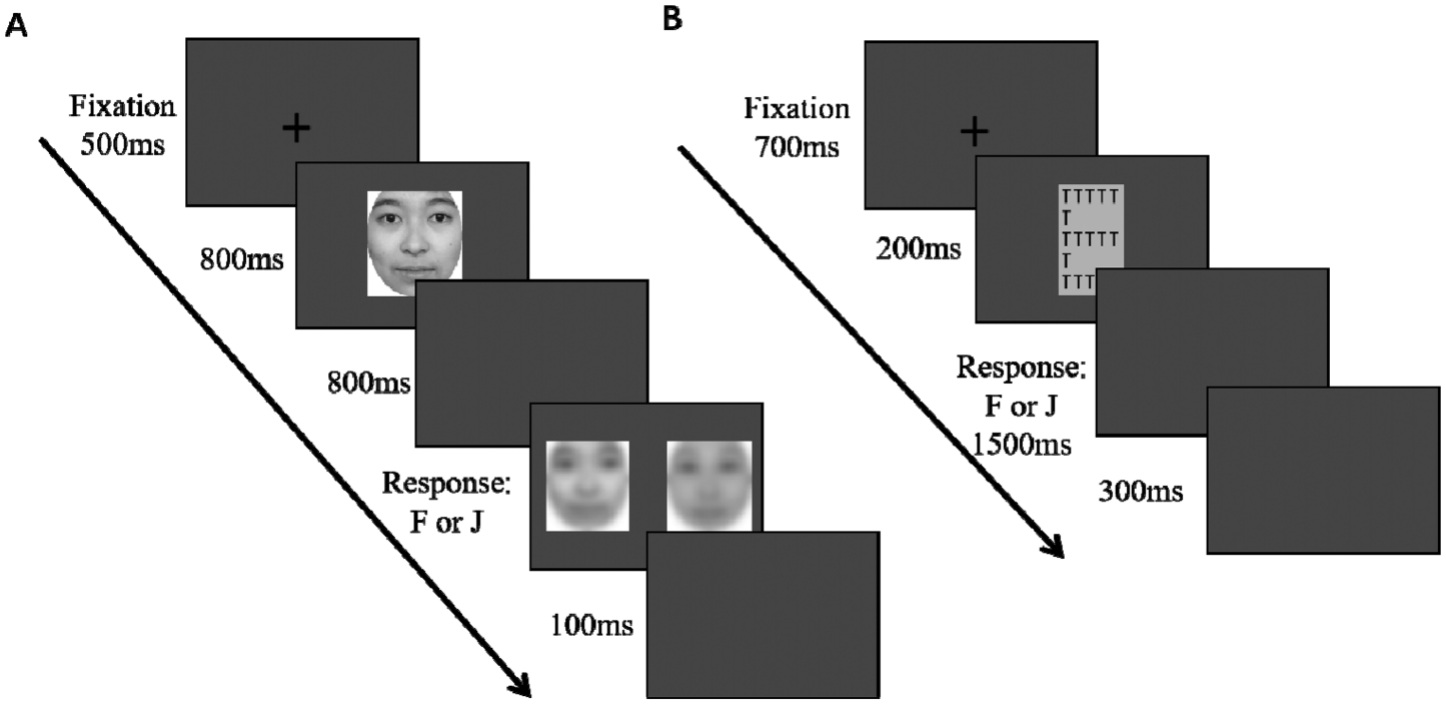
Example trials for blurred face delayed matching task (A) and Navon task (B).

Task 4 was the Navon task. All 91 participants performed Tasks 2. They then sat comfortably in a chair with their face positioned 53 cm away from a 14-in. computer screen, which had a resolution of 1920 × 1280 and a refresh rate of 60 Hz. The other environments were the same as those in Task 1. All participants took part in the Navon task, in which they reported whether the target stimulus (E) was present at the global or local level by pressing a key. The task had two blocks, with each block containing 120 trials, including 60 trials for the target stimulus and 60 trials for the non-target stimulus. For both types of stimuli, the number of global and local stimuli was identical. Each trial was initiated by a black “+” sign displayed at the center of the gray screen for 700 ms. Subsequently, a stimulus appears for 200 ms, followed by a blank white screen for 1500 ms. Participants were instructed to judge as quickly as possible whether it was the target stimulus within 1700 ms. Each response was followed by a 500-ms blank screen and the next trial. Figure 2 shows example trials of Tasks 3 and 4.

### Data Processing and Analysis

In Task 2, scores greater than 1 for common objects may have resulted from response bias. Correlation analysis revealed a significant positive association between participants’ face-likeness ratings for common objects and face-like objects (*R* = 0.514, *p* < .001), suggesting that some participants exhibited a response bias. Specifically, a desire to please the experimenter may have led them to assign higher ratings to all images. To account for individual response bias, each participant's mean face-likeness rating for common objects was used as an individual baseline. This baseline was then subtracted from their ratings of face-like objects to produce adjusted scores, ensuring a more accurate reflection of their perception for further analysis. A regression analysis was performed using the mean ratings of all face-like objects (with the baseline subtracted) and the reported face ratings from Task 1.

Prior to further analysis, we calculated the accuracy for Task 3. Data from one participant whose accuracy was below the random chance level (50%) were excluded. Thus, data from 69 participants (33 men and 36 women) were included in the formal analysis.

## Results

**Figure 3.**
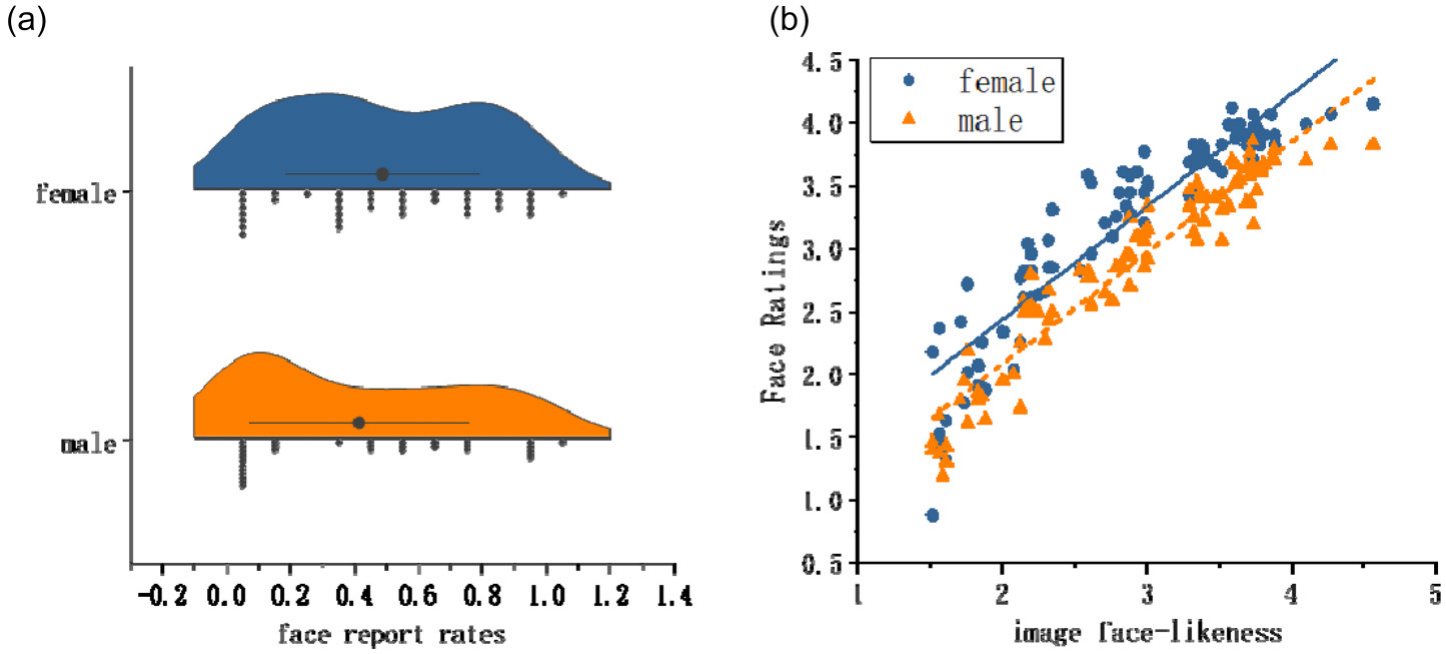
(a) Raincloud plots showing face pareidolia rates in male (top, blue) and female (bottom, orange) participants; (b) regression analysis results of face ratings (upright) and image face-likeness in women (blue blobs) and men (orange triangles).

In Task 1, A Mann–Whitney U test was conducted to compare the face report ratios between males and females. The results indicated no significant difference between groups, *U* = 521.00, *Z* = −1.083, *p* = .279. The mean face report rates by gender are presented in Figure 3a.

**Figure 4.**
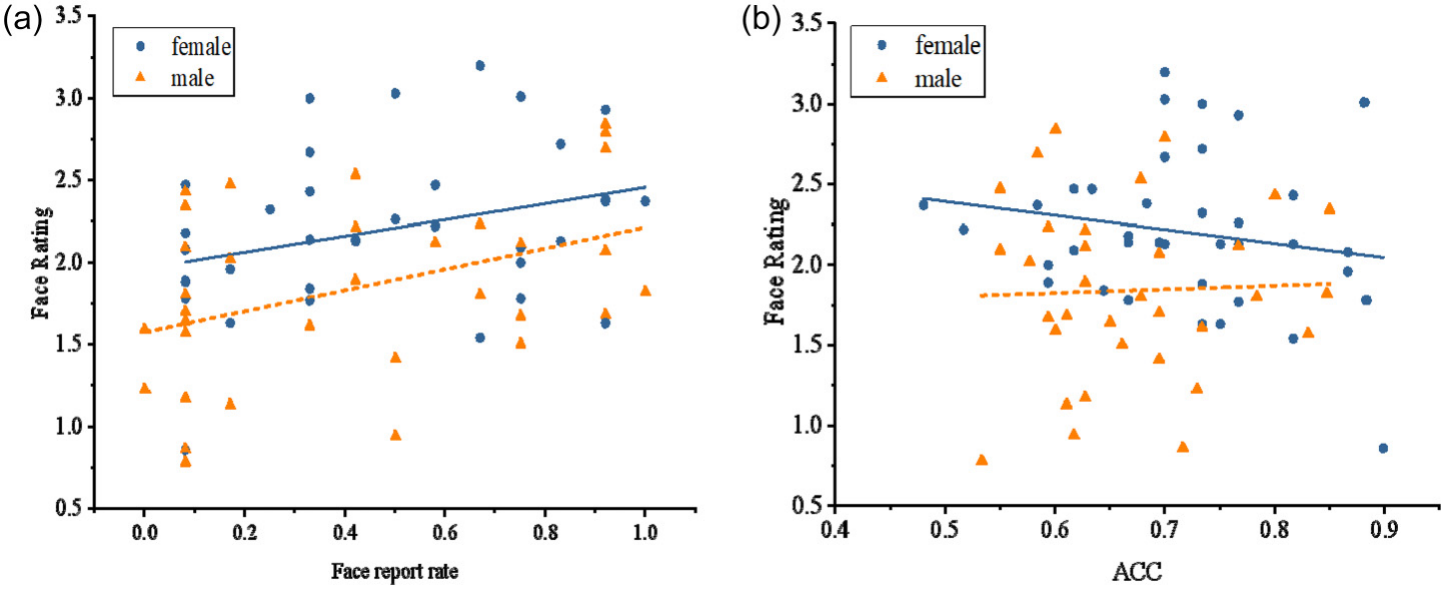
(a) Regression analysis results of face ratings (upright) and face report rates in women (blue blobs) and men (orange triangles); (b) regression analysis results of face ratings (upright) and accuracy rate (ACC) of the delayed matching task in women (blue blobs) and men (orange triangles).

In Task 2, there were no significant gender differences in face ratings for common objects, *t*(68) = 1.343, *p* = .184. To examine the effects of image face-likeness and gender on participants’ ratings, a linear mixed-effects model (LMM) was conducted. Image face-likeness (continuous) and gender were entered as fixed effects, along with their interaction term. Random intercepts were specified for participants and images to account for subject- and stimulus-level variability. The results showed a significant main effect of gender, *b* = 0.351, *SE* = 0.143, *t*_[142.9]_ = 2.45, *p* = .016, indicating that female participants gave higher ratings than male. Image face-likeness also had a significant main effect, *b* = 0.902, *SE* = 0.054, *t*(595.1) = 16.63, *p* < .001, with more face-like images receiving higher ratings overall. However, the interaction between gender and image face-likeness was not significant, *b* = –0.003, *SE* = 0.028, *t*(6140) = –0.10, *p* = .924, suggesting that the effect of face-likeness on ratings did not differ by gender. Random effects analysis showed significant variability across participants (variance = 0.24, *p* < .001) and images (variance = 0.05, *p* < .001), justifying the inclusion of random intercepts in the model. Figure 3b shows fitted regression lines for the relationship between image face-likeness and ratings by gender (Figure 4).

Combining the results of Tasks 1 and 2, we found that gender differences appeared only in the face-likeness rating task but not in the spontaneous face detection task. To better distinguish the effects of different task demands, a multiple linear regression was conducted to predict face-likeness ratings from face report rates, gender, and their interaction.The overall model was significant, *F*(3,66) = 6.643, *p* = .001, *R*^2^ = 0.231. There was a significant main effect of face report rates, *β* = 0.568, *t*(66) = 3.108, *p* = .003, indicating that performance on the face report task positively predicted face-likeness ratings. A significant main effect of gender was also observed, *β* = −0.298, *t*(66) = −2.758, *p* = .007, suggesting that women perceived more face-likeness than men. However, the interaction between gender and face report rates was not significant, *β* = 0.043, *t*(66) = 0.397, *p* = .692, indicating that the predictive effect of face report rates on face-likeness ratings did not differ by gender.

An independent-sample *t*-test was performed to determine the accuracy rate of Task 3. The results showed that the accuracy rate of women (0.72 ± 0.09) was higher than that of men, 0.66 ± 0.08, *t*(68) = −2.56, *p* = .012, indicating that women's ability to process facial configuration was higher than that of men. Taking the reaction time of correct judgement as the dependent variable, the results revealed no significant differences in reaction time between genders, *t*(68) = −0.99, *p* = .325, *M* female = 2147 ± 740 ms, *M* male = 1975 ± 701 ms. A multiple linear regression was conducted to examine whether accuracy in the blurred face matching task, gender, and their interaction predicted face-likeness ratings. The main effect of accuracy was not significant, *β* = −0.059, *t*(65) = −0.470, *p* = .639, indicating that the ability to process facial configurations did not predict face-likeness ratings. Additionally, the interaction between accuracy and gender was not significant, *β* = 0.102, *t*(65) = 0.807, *p* = .422.

In Task 4, an index of perceptual preference (*I*) of each participant was calculated to identify differences in global and local processing tendencies between participants, as follows:

I=MeanRT(Local)−MeanRT(Global).

The reaction times used in this calculation were based only on correct responses. Smaller *I* values indicated a greater inclination for local processing, and vice versa (Boccia et al., 2014).

**Figure 5.**
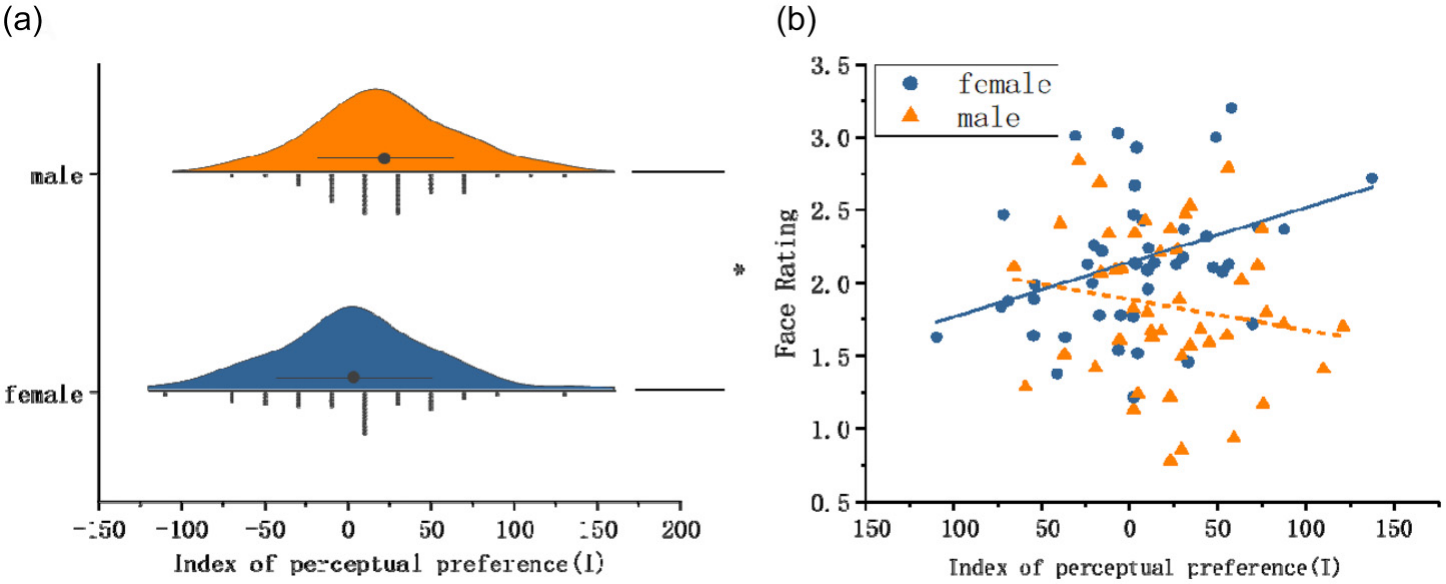
(a) Raincloud plots illustrating the global-local perceptual index (*I*-score) from the Navon task, separately for female (top, orange) and male (bottom, blue) participants. (b) Results of the regression analysis between face-likeness ratings (upright condition) and the global-local perceptual index (*I*-score) in women (blue blobs) and men (orange triangles). A stronger preference for global processing was associated with higher face-likeness ratings in females, but not in males.

In the Navon task, with gender as an independent variable, the independent sample *t*-test on the index of perceptual preference (*I*) showed that the index of men (*M* = 25.73 ± 39.44) was significantly higher than that of women (*M* = 5.12 ± 48.58, *t*(75) = −2.04, *p* = .045, Cohen's *d* = 0.47). This suggested that men were more likely than women to view stimuli as a whole (Figure 5a). To examine the influence of perceptual preference, a regression analysis of the face-likeness ratings on the index of perceptual preference (*I*) was conducted for women and men (Figure 5b). A multiple linear regression was conducted to examine whether the index of perceptual preference (*I*), gender, and their interaction predicted face-likeness ratings. The overall model was significant, *F*(3,87) = 4.882, *p* = .003, *R^2^* = .144. A post hoc power analysis using G*Power 3.1 indicated that with a sample size of 91, α = .05, three predictors, and *R²* = .144, the achieved power was 0.85. The main effect of the index of perceptual preference was not significant, *β* = 0.060, *t*(87) = 0.590, *p* = .556, indicating that the index (*I*) could not predict face pareidolia. However, the interaction between gender and the index of perceptual preference was significant, *β* = −0.235, *t*(87) = −2.299, *p* = .023, specifically, perceptual preference positively predicted face-likeness ratings in females, *β* = 0.296, *t*(87) = 2.203, *p* = .030, but not in males, *β* = −0.175, *t*(87) = −1.132, *p* = .260.

## Discussion

This study explored factors affecting gender differences in face pareidolia using a series of experiments. The results demonstrated that women were more likely to perceive objects as faces in face-likeness rating task, and this difference was not influenced by facial configuration processing. Moreover, global processing tendency for common objects predicted the extent of face pareidolia in women but not in men.

### Gender Differences and Task Requirements

Previous studies have found that visual input is regulated by top-down information, particularly fuzzy stimuli (Bar et al., 2006). In the present study, task requirements played an important role in gender differences in face pareidolia; when face-likeness ratings was required, women showed more face pareidolia than men, which was consistent with previous research findings (Proverbio & Galli, 2016). Although no gender differences were observed in the spontaneous reporting task, the regression analysis showed that the reporting rate score predicted face-like ratings. Notably, gender differences were observed only in the explicit detection task, suggesting that gender differences in face pareidolia may be more likely to emerge when face detection is explicitly required, though further evidence is needed to clarify this possibility. The unique facial processing system may be triggered by a top-down mechanism; hence, face-related information in subsequent visual input would receive more attention (Ge et al., 2006), enhancing the coding of face information (Akechi et al., 2014; Wild & Busey, 2004).

However, the present study's findings also differed from those of some previous studies (Pavlova et al., 2020; Wardle et al., 2022), which reported no significant gender differences in face pareidolia. This discrepancy may stem from differences in stimuli or task design. Pavlova et al. (2020) found that enhanced facial perception in women emerged only under the inverted condition, suggesting that men may require more configurational cues to perceive objects as faces.

Moreover, unlike previous studies (e.g., Proverbio & Galli, 2016), we derived face-likeness scores by subtracting ratings for common objects from those for face-like objects, thereby reducing potential bias from participants’ tendency to comply with perceived experimental demands. In addition, gender differences were observed across objects with varying levels of face-likeness, but not in response to common objects. Therefore, the gender difference observed was not caused by women giving high scores to all objects to meet the requirements of the experiment; rather, it was because those objects appeared more face-like to them.

### Face-Specific and Common Object Processing

Our results regarding face-specific processing were consistent with those of previous studies (McBain et al., 2009). Women's performance on the delayed blurred face-matching task was superior to that of men, indicating that women had an advantage over men in terms of facial configural information processing (McBain et al., 2009; Rehnman & Herlitz, 2006). However, this advantage did not lead to increased face pareidolia. The ability to extract facial configuration information did not predict face pareidolia in either men or women. Therefore, the observed gender difference in face pareidolia cannot be attributed to differences in facial configuration processing advantage. Moreover, previous studies on face pareidolia did not report gender differences in facial configuration processing as reflected by N170 responses or fusiform face area (FFA) activation(Akechi et al., 2014; Hadjikhani et al., 2009; Liu et al., 2014; Proverbio & Galli, 2016). Therefore, processing facial configuration information and recognizing stimuli as a face reflect different cognitive processes. The former mechanism distinguishes the target face from other faces by processing global structural information (Wang et al., 2012), while the latter separates face-like objects from the surrounding environment (Tsao & Livingstone, 2008). This segregation process relies not only on the holistic processing of face-like structures, but also on whether the stimulus matches an internal face template—an evaluation influenced by top-down facial criteria (Smith et al., 2012). Before facial configurations and local features can be further processed, the object must first be categorized as a face. Face-specific processing, which demands considerable cognitive resources, is only engaged when the stimulus exceeds the perceptual threshold for face-likeness (Tsao & Livingstone, 2008).

The distinction between faces and common objects may occur at an earlier stage and is primarily affected by the processing of common objects rather than faces (Wardle et al., 2020). Previous studies have shown that only the ability of facial configuration processing—and not the cognitive style of common objects—can predict facial recognition (Wang et al., 2012). In addition, individuals who lack the ability to recognize faces, such as those with prosopagnosia, can still activate face-related brain areas and perceive nonexistent faces in noisy pictures under the influence of top-down information (Righart et al., 2010). This indicates that the two processes occur at different stages. Thus, face pareidolia may not be a form of facial processing but rather a type of anthropomorphic perception of face-like objects.

In line with this thinking, we examined the effect of cognitive style, and our results showed that although global processing was stronger in men, aligning with previous findings (Razumnikova & Volf, 2012; Roalf et al., 2006), face pareidolia was not. Global processing alone was not sufficient to recognize an object as a face. While global processing alone was not sufficient for an object to be recognized as a face, women with a global perceptual style were more likely to perceive objects as faces—possibly due to the combined influence of global processing bias and more liberal face detection criteria.

This differs from the results of Boccia et al. (2014), who found that the global processing tendency facilitated a clear evaluation of Arcimboldo portraits (required to judge whether the stimulus is clear or ambiguous) in both men and women. This might be attributable to different task requirements and materials used. Unlike Arcimboldo portraits, which have no alternative interpretation except for faces when processed as a whole, the present study used daily objects and real scenes. Even if they were processed as a whole, they had “literal” meanings other than faces. For instance, for a face-like house, participants who use global processing focus on its overall face-like structure and classify it as either a face or a house.

### Judgment Criteria

In summary, the observed gender differences in face pareidolia could not be adequately accounted for by visual processing factors. Instead, they may be partially explained by top-down influences, whereby women were more likely than men to interpret objects with face-like structures as faces. In other words, gender differences appeared in the criteria for what constitutes a face, rather than in sensitivity to visual information. Previous studies have found that gender differences in processing face-like objects are reflected in vertex positive potential (VPP), which is influenced by the mental imagery of a face (Lu et al., 2017; Proverbio & Galli, 2016). This suggests that gender differences in face pareidolia may be due to top-down modulation. A preference for global processing was found to cause both men and women to detect T-shaped or inverted triangular structures in face-like objects (Tsao & Livingstone, 2008), which is the prerequisite for face pareidolia. Men could more easily detect global structures than women; however, global processing itself is insufficient for recognizing objects as faces, as men had more stringent criteria than women regarding what constitutes a face, requiring more visual evidence (Pavlova et al., 2020).

An important question is why women have more relaxed criteria for what constitutes a face. As the most common social stimulus, faces are separated from the processing of common objects by the visual system (Ge et al., 2006; Leibo et al., 2011) to facilitate the extraction of social information, such as gender, expression, and direction of gaze. From an evolutionary perspective, detecting and distinguishing faces from other objects in the surrounding environment is critical for survival (McKone et al., 2007). Rapid detection of faces is necessary in certain situations, such as fear, anxiety, and lack of control, even considering the risk of false positives (Cataldo & Cohen, 2015; Epley et al., 2008; Whitson & Galinsky, 2008). Pareidolia faces have a similar visual search advantage (Keys et al., 2021; Takahashi & Watanabe, 2015), emotional processing (Alais et al., 2021), and gaze direction (Takahashi & Watanabe, 2013) as real faces, suggesting that this process is beyond the simple detection of a face pattern. Both real and pareidolia faces activate the FFA and advanced areas, such as the limbic system (Kosaka et al., 2003), as well as social and emotional regions in the superior temporal sulcus (Rossion et al., 2011). Therefore, face pareidolia serves not only to detect familiar facial patterns but also to identify potential individuals in the environment.

However, women pay more attention than men to social stimuli (Alaerts et al., 2011; Proverbio et al., 2008), including people and faces (Proverbio et al., 2008), as well as the direction of others’ gazes (Alwall et al., 2010; Bayliss et al., 2005). Compared with men, women rate both real faces and face-like objects as more likable (Pavlova et al., 2016; Proverbio, 2017). Women's brains are reported to be more responsive to face-like objects, with greater activation in relevant areas of the social brain (Proverbio & Galli, 2016). This indicates that faces, including pareidolia faces, are a special stimulus that can arouse women's social interest. Although no gender differences were found in the evaluation of emotional expression, age, and gender in pareidolia faces, both men and women tended to interpret inanimate objects as angry male faces (Wardle et al., 2022). Such a scenario may be more threatening for women than for men, with threatening faces emerging faster in consciousness (Abir et al. 2018). Consequently, women obtain more social information from face-like objects, leading to false positives under less strict criteria. Thus, women focus more on social information and are more likely to regard nonsocial stimuli as social stimuli (such as faces) than men. These findings shed light on gender differences in social cognition.

### Implications for Future Research

This study explores the underlying factors contributing to gender differences in face pareidolia and reveals that this phenomenon, occurring in early processing, may be influenced by social information. However, more direct evidence from behavioral and neuroimaging studies is needed to further substantiate these findings. Previous researchers have suggested that face pareidolia may be affected by social motivation (Akechi et al., 2014), such as prioritizing social information, expecting social cooperation, and interacting with others (Chevallier et al., 2012). Studies have found that social motivation causes participants to anthropomorphize nonliving objects (Epley et al., 2008) and affects the judgment of the animacy of toy faces. Based on a large number of studies on autism, Baron-Cohen (2004) proposed the extreme male brain theory of autism, noting that empathy was lower in men than in women and that empathy among patients with autism was lower than that among typically developed people. Individuals with autism have a low rate of face pareidolia (Pavlova et al., 2017), similar to gender differences observed in typically developed individuals. Therefore, whether social motivation contributes to lower rates of face pareidolia in men and people with autism remains unclear. Future research should explore the effects of top-down social information on face detection and of social information on the processing of fuzzy visual information. Moreover, the experimental paradigm used in the present study has certain limitations, particularly in its inability to directly assess individual differences in face detection criteria. Future research could consider employing paradigms based on signal detection theory (SDT) to disentangle perceptual sensitivity from decision criterion, which would be essential for understanding the underlying mechanisms of individual differences in face pareidolia. In addition, Task 3 may be influenced by general pattern-matching processes. Although the task was based on previous studies, its use of same-view, low-pass filtered images limits its ability to accurately assess individual differences in configural face processing. Future research could adopt more refined paradigms, such as identity matching across different viewpoints or the SF bubbles method, to better understand how sensitivity to specific spatial frequency information in faces contributes to pareidolic perception.
